# Seroprevalence of hepatitis C and associated risk factors in urban areas of Antananarivo, Madagascar

**DOI:** 10.1186/1471-2334-8-25

**Published:** 2008-02-29

**Authors:** Charles E Ramarokoto, Fanjasoa Rakotomanana, Maherisoa Ratsitorahina, Vaomalala Raharimanga, Richter Razafindratsimandresy, Rindra Randremanana, Mala Rakoto-Andrianarivelo, Dominique Rousset, Voahangy Andrianaja, Vincent Richard, Jean-Louis Soares, Leon P Rabarijaona

**Affiliations:** 1 Unité d'Epidémiologie, Institut Pasteur de Madagascar, BP 1274, Antananarivo 101, Madagascar; 2 Laboratoire de virologie, Institut Pasteur de Madagascar, BP 1274, Antananarivo 101, Madagascar

## Abstract

**Background:**

The risk factors for the transmission of HCV vary substantially between countries and geographic regions. The overall prevalence in south and east Africa region has been estimated to be 1.6% but limited information about the epidemiology of HCV infection in Madagascar is available

**Methods:**

A cross-sectional survey for hepatitis C antibodies was conducted in 2,169 subjects of the general population of Antananarivo to determine seroprevalence of hepatitis C and associated risk factors.

**Results:**

The overall seroprevalence was 1.2% (25/2,169). The prevalence did not differ significantly according to gender but it increased with age (Chi2 tendency test, p < 10^-5^). The variable history of hospitalization, previous therapeutic injections, dental treatment, intravenous drug use, and abnormal ALT and AST were statistically significantly related with the presence of HCV antibodies. No relationship with past history of blood transfusion was observed.

**Conclusion:**

HCV prevalence in Madagascar seems to be similar to that in most other east African countries. Age appears to be an important risk factor. Iatrogenic causes of HCV transmission need to be further evaluated because all HCV cases had a history of receiving therapeutic injections and data suggested a cumulative effect in relation with therapeutic injections.

## Background

Hepatitis C virus (HCV) is a major cause of chronic liver disease worldwide. In 1999, the WHO estimated a worldwide prevalence of about 3%, with the virus affecting 170 million people [1]. The major channels for HCV transmission are all related to exposure to blood and blood products. The risk factors for the transmission of HCV vary substantially between countries and geographic regions. In Europe, the general prevalence of antibodies to HCV (anti-HCV) is about 1% [2]but varies between countries (0.87% in Belgium [3], 3.2% in Northern Italy [4], 1.3% in France [5,6]). In Central and South America, the prevalence varies between 1.2% [7] (Puerto Rico) and 6.3% [8] (Mexico). Intermediate rates of HCV infection have been reported across Asia (0.49% in Japan [9], 1% in China [10,11]) with higher rates in some areas (Hubei province – 30.1%, Inner Mongolia Autonomous Region – 31.9% [12]). The recently estimated prevalence in Australia is 2.3% [13]. There have been fewer studies in Africa; however, the region is reported to have the highest HCV prevalence rate (5.3%) [1] with differences between regions [14]. Prevalence is generally high in central Africa (6.0%) [14] with the maximum in Cameroon (13.8%) although lower rates have been reported (1.9% in pregnant women) [15]. Nevertheless, much higher rates (20%) have been found in Egypt [16-18]. In west Africa, the overall weighted average prevalence is 2.4% [14]. The highest HCV prevalence for this region is in Guinea (5.5%) [14]. The overall prevalence in south and east Africa region has been estimated to be 1.6% [14]: among blood donors 1.6% in Ethiopia and 0.9% in Kenya [19,20]. The lowest HCV prevalence for this region is in South Africa (0.1%) [14].

Only limited information about the epidemiology of HCV infection in Madagascar is available. Previous studies have suggested a low prevalence of HCV seropositivity among a sample of patients in rural areas [21-23]. Although HCV infection has been identified as one of the major causes of chronic hepatitis and hepatocellular carcinoma in Antananarivo [24], its prevalence in the population and routes of transmission are largely unknown. The aim of this study was to determine the prevalence of anti-HCV antibodies and the possible risk factors for transmission in the general population of Antananarivo.

## Methods

### Study design and setting

Antananarivo (*Commune Urbaine d'Antananarivo or CUA*) is the capital city of Madagascar. It is located on the central highlands. The CUA had a population of about 1.5 million in 2004 (report from the Mairie d'Antananarivo-Ville). Antananarivo consists of administrative, commercial, industrial and residential areas, with patches of agricultural land that are mostly rice fields. The city is divided into six administrative districts.

The study was carried out during March and May 2004, and targeted the general population of the CUA. All inhabitants older than 2 years living in the study site were eligible to participate in the survey. A cross-sectional study design was used to achieve the primary objective: to estimate the prevalence of HCV seropositivity and evaluate potential risk factors by comparing HCV seropositive and seronegative individuals. A two-stage cluster sampling technique, according to the Expanded Programme on Immunization (EPI) cluster survey approach, was employed to obtain the required sample. A cluster was defined as a "Fokontany", the smallest administrative unit. The sampling unit was a household randomly selected within each cluster.

### Sample size

Using EPI Info software and assuming a HCV seroprevalence of 1.2% in the study population with 95% confidence level, error limits of +/-1% and a design effect of 2, the estimated sample size required was 2234. However, the target sample size was increased to 2310, made up of 70 clusters each with 33 subjects.

### Data collection and serology

Physicians from the Ministry of Health of Madagascar and Pasteur Institute of Madagascar went to the selected household and approached the head of the family. They explained the purpose and objectives of the study and asked for written informed consent (from participants or parents for minors) before administering the questionnaire and collecting a blood sample. The team administered a pre-tested questionnaire to the participant to obtain information on socio-demography and potential risk factors for HCV transmission. The questions in the questionnaire gathered demographic characteristics including age, sex, socio-economic status, matrimonial status (this variable was classified as subjects "living as a couple, married or not" and "single"), and also the following information: number of years in education, number of therapeutic injections, history of surgical procedure, especially circumcision for men, transfusion with blood or blood products, history or current use of intravenous drugs, history of tattooing or scarification, history of jaundice, history of HBV immunization and history of traditional medicine use.

Each blood sample was analyzed first with a third generation ELISA, Monolisa^® ^anti-HCV Plus version 2, (BioRad, Marne-La-Coquette, France) according to the recommendations of the manufacturer. Positive sera were retested using an immunoblot test, Deciscan^® ^HCV Plus assay (Biorad, Marne-La-Coquette, France) to confirm the results. All Monolisa-positive samples and pools of 10 negative sera were screened for the presence of viral RNA using reverse transcription polymerase chain reaction amplifying the 5'untranslated region (UTR). The genotype was determined using the BLAST tool with another 5'UTR fragment as described previously [25]. If results with both the Monolisa test and the Deciscan were positive, the subject was considered to be seropositive for HCV.

### Data management and analysis plan

EPI Info version 6 was used for data entry [*EPI Info: a word processing database and statistics program for public health; Version 6*. Atlanta, GA: Center for Disease Control and Prevention; 1995]. The data was analyzed using the Statistical Package for Social Sciences (SPSS) version 11.5 [SPSS. *Statistical Package for Social Sciences; Version 10.0*. Chicago, IL: SPSS Inc; 1996]. The analysis was carried out at three levels: descriptive analysis, and univariate and multivariate analyses. Descriptive statistics of socio-demographic variables and other characteristics of the sampled population were computed. Means and standard deviations (SD) were calculated for quantitative variables and proportions for categorical variables. Logistic regression analysis was performed to measure the association between outcome and each independent variable. Odds ratios (OR) and 95% confidence intervals (CI) were calculated from β coefficients and their standard errors. Associations between independent variables were assessed using appropriate tests before performing multivariate analysis. A multivariate logistic regression model was employed with stepwise backward elimination of non-significant variables, with HCV antibody status as the dependent variable. P values < 0.05 were considered to be statistically significant.

### Ethical clearance

The study was approved by the Ministry of Health and the National Ethics Committee of Madagascar. Permission and informed consent were obtained from the Antananarivo district and the participants of the study.

## Results

Among the 2,310 subjects contacted for the study, 2,169 older than two years (94%) agreed to participate and had serum tested for HCV antibody. Most subjects who were informed of the study agreed to participate, the exact number of subjects who refused participation was not determined. The mean age of the 2169 subjects was 29.1 years (CI95% [28.3–29.8]). The sex ratio was 0.62 (834 males/1335 females).

Of the 2,169 sera collected, 36 (1.6%) scored positive in the Monolisa anti HCV test version 2. The 36 positive samples were re-tested using Deciscan HCV Plus: 25 were confirmed positive for anti-HCV (1.2% with 95% CI [0.75–1.72], eight were indeterminate and three negative. Of the 36 subjects positive for anti-hepatitis C antibody (Monolisa), 17 (47.2%) were positive for HCV-RNA (16 positive and 1 indeterminate by Deciscan). Thus, the prevalence of active HCV infection in the study population was 0.8% (17/2169). Nine of the positive samples (52.9%) contained genotype 1 (subtype 1b) HCV, and eight genotype 2 (47.1%).

The prevalence was not significantly different according to gender (males: 1.1% (9/834), females: 1.2% (16/1335); p = 0.8) but HCV seropositivity and HCV RNA positivity increased with age (Table 1) (Chi2 tendency test, p < 10^-2^). There were no other statistically significant differences between the anti-HCV positive and anti-HCV negative groups in terms of demographic characteristics or ritual practices including circumcision and scarification (Table 2). Of note, male circumcision was a frequent ritual practice (96.4%). Thirteen of the 25 subjects with anti-HCV, but only 692 of 2142 anti-HCV negative subjects, reported a history of hospitalization (p = 0.04).

**Table 1:** Seroprevalence of anti-Hepatitis C and risk factors according to age group among Antananarivo inhabitants in 2004.

	**Age group **(years)				**P value**
	0 – 24 yrs (n = 1018)	25 – 44 yrs (n = 688)	45 – 64 yrs (n = 372)	> 64 yrs (n = 91)	
**Anti-HCV positive**	1 (0.1)	7 (1.2)	10 (2.7)	7 (7.7)	<10^-2^
**HCV RNA positive**	0 (0.0)	2 (0.3)	8 (2.2)	7 (7.7)	<10^-2^
**Previous therapeutic injections**	756 (74.3)	607 (88.3)	348 (93.5)	86 (94.5)	<10^-2^
**Previous blood transfusion**	11 (2.1)	52 (7.6)	58 (15.6)	10 (10.9)	<10^-2^
**Previous dental treatment**	442 (43.5)	528 (76.8)	300 (81.1)	66 (72.5)	<10^-2^
**ALT abnormal**	20 (1.9)	17 (2.5)	11 (2.9)	2 (2.2)	<10^-2^
**AST abnormal**	152 (14.9)	63 (9.1)	53 (14.2)	11 (12.1)	<10^-2^

**Table 2:** Demographic features and risk factors associated with seroprevalence of anti-Hepatitis C (Univariate analysis) among Antananarivo inhabitants in 2004.

		**Anti-hepatitis C antibody**		
**Risk factor**		Positive n (%)	Negative n (%)	P value

**Matrimonial status**	In couple	18 (75.0)	977 (61.6)	
	Single	6 (25.0)	609 (38.4)	0.17
**Educational level**	Educated	17 (68.0)	1259 (58.7)	
	Non-educated or low level	8 (32.0)	885 (41.3)	0.35
**Circumcision**	No	0 (0.0)	30 (3.6)	
	Yes	9 (100.0)	795 (96.4)	0.99
**Previous scarification**	No	24 (96.0)	2109 (99.2)	
	Yes	1 (4.0)	17 (32.3)	0.19
**Previous hospitalisations**	No	12 (48.0)	1452 (67.7)	
	Yes	13 (52.0)	692 (32.3)	0.04
**Previous jaundice**	No	23 (92.0)	1968 (92.7)	
	Yes	2 (8.0)	155 (7.3)	0.70
**Previous therapeutic injections**	No	0 (0.0)	371 (17.3)	
	Yes	25 (100.0)	1772 (82.7)	0.01
**Previous surgical operation**	No	17 (70.8)	1808 (84.6)	
	Yes	7 (29.1)	330 (15.4)	0.08
**Blood transfusion**	No	21 (84.0)	2017 (94.1)	
	Yes	4 (16.0)	127 (5.9)	0.06
**Dental treatment**	No	3 (12.0)	825 (38.6)	
	Yes	22 (88.0)	1314 (61.4)	<10^-2^
**Intravenous drug use**	No	21 (95.5)	2046 (99.8)	
	Yes	1 (4.5)	3 (0.2)	0.04
**Tattooing**	No	20 (86.9)	1874 (89.4)	
	Yes	3 (13.1)	222 (10.6)	0.45
**Aminotransferase (ALT)**	Normal	22 (88.0)	2055 (97.7)	
	Abnormal	3 (12.0)	47 (2.3)	0.02
**Aminotransferase(AST)**	Normal	16 (64.0)	1832 (87.1)	
	Abnormal	9 (36.0)	270 (12.9)	<10^-2^

The percentage of individuals having received previous dental treatment was significantly higher among anti-HCV positive (88%) than among anti-HCV negative (61.4%) subjects (p < 10^-2^). The percentage of individuals with a history of blood transfusion was not significantly different between the anti-HCV positive (16.0%) and anti-HCV negative (5.9%) groups.

All HCV-positive subjects had received therapeutic injections whereas none of those who did not receive an injection was anti-HCV positive. The proportion of anti-HCV positives increased with the number of therapeutic injections (Chi2 tendency test, p < 10^-2^): 0.7% (8/1053) between 1 to 10 injections, 0.9% (2/221) between 11 to 15 injections and 2.7% (14/521) for more than 15 injections.

Few subjects (4/2067) reported intravenous drug use: 1/22 subjects with HCV antibodies and 3/2049 anti-HCV negative subjects (p = 0.04); intravenous drug use was significantly associated with anti-HCV positivity (Table 2).

Finally, as expected, high aminotransferase (ALT and AST) activity was significantly associated with anti-HCV positivity, and hepatic enzyme (ALT, AST) activities were high in the 17 subjects with active HCV infection.

The major channels for HCV transmission (blood transfusion, therapeutic injections, dental treatment) are all related to age and increased with age up to the [45–64 yrs] age group (Table 1).

In the multiple logistic regression model with backwards elimination, only age and aminotransferase (AST) were independently associated with a positive HCV antibody result (Table 3).

**Table 3:** Independent risk factors associated with seroprevalence of anti-Hepatitis C among Antananarivo inhabitants identified by multiple logistic regression, Antananarivo, 2004.

**Risk factor**	**Adjusted odds ratio**	**95% confidence interval (CI)**	**P value**
**Age**			
0 – 24	1.0	(1.3 – 85.3)	0.02
25 – 44	10.4	(3.6 – 220.5)	<10^-2^
44 – 64	28.1	(10.3 – 697.7)	<10^-2^
65 & more	84.7		
			
**Aminotransferase (AST**)			
Normal	1.0		
Abnormal	4.4	(1.8 – 10.3)	<10^-2^

## Discussion

This survey indicates that the seroprevalence of HCV antibodies in Antananarivo is low (1.2%) and similar to values reported by previous studies in Madagascar. In 1994, Zeller *et al*. found a prevalence of 1.2% in the general population of Antananarivo and Toamasina provinces [23]. This prevalence is similar to rates reported for the south and east of Africa (1.6% for the general population, 1.9% for blood donors) [14]. In this region, Mozambique (2.8%) and Tanzania (3.2%) have the highest HCV prevalence. The prevalence in Madagascar is lower than the sub-Saharan African median of 2.2% [14].

We have no information about the subjects who refused to participate in the study, and this may limit the representativeness of the sample. Other limitations of the study are linked to the difficulties inherent in self-reporting of behaviour, including in particular drug use, to the lack of clinical information (acute or chronic liver disease, dialysis) and of individual information for some of the risk factors.

In the developing world, which includes Madagascar, the prevalence of HCV is thought to be both higher and distributed more generally throughout the adult population than in industrialised countries [1]. Given the low transmission of HCV through sexual contact in sub-Saharan Africa [14] and rarity of intravenous drug use in Madagascar, the observed increase of anti-HCV seroprevalence with age is suggestive of unsafe medical practices (unsterile injections and other iatrogenic routes of transmission) as the main risk factor for HCV infection. In the *Commune Urbaine d'Antananarivo *(CUA), all cases had a history of previously receiving therapeutic injections and the analysis of risk factors according to age group possibly suggests a cumulative effect: the risk increases with the number of therapeutic injections.

Enzyme immunoassays and PCR assay indicated increasing prevalence with age, suggesting that there may be ongoing and repeated exposures to HCV infection, possibly through the medical care system. However, a large proportion of anti-HCV seronegative individuals had also had injections. Moreover, not only HCV prevalence increased with age, but also the blood exposure risk. (*Moreover, both HIV and blood exposure risk increased with age.)*

Univariate analysis showed that dental treatment and previous injections increased the risk HCV seropositivity. This could be explained by the lack of prevention in healthcare (poor sterilization procedures, unsafe injections) in Madagascar. Until the early 1990s, most primary health care programs were supplied with glass or plastic syringes designed to be washed and sterilized between uses. Earlier, boiling was deemed acceptable for decontaminating these syringes, but evidence on the thermostability of resistant spores and hepatitis viruses prompted a change to steam sterilizers or pressure cookers. These decontamination procedures were highly dependent upon the availability of fuel for heating, regular maintenance of the sterilizers, availability of spare parts, well-trained health care workers, and good management and control procedures. Lack of one or more of these essential conditions often resulted in the use of contaminated injection equipment. The introduction of disposable syringes and needles designed for single use tended to exacerbate the problem because the conditions and culture of scarcity still prevailed; disposable products could be easily reused, and they were not designed to be cleaned and sterilized.

Half of the more than 16 billion injections administered in the developing world have been estimated to be unsafe [26]. More recently, health officials estimate that annually, the re-use of injection equipment may cause 20 million infections with hepatitis B virus, 2 million infections with hepatitis C virus and 250,000 infections with HIV worldwide [27].

It should be stressed that in our study, the prevalence of HCV infection was not associated with blood transfusion. This observation is surprising in view of the recent introduction (around 2000, report from MOH) of anti-HCV screening tests in hospital blood centres in the CUA. Presumably, the reservoir levels of the virus in blood in the CUA are low. But this observation concerning blood transfusion contrasts with many studies elsewhere in the world^6^, and also with a previous survey (in 1994) of the general population in two provinces of Madagascar where a significant relationship was found between history of blood transfusion and HCV infection [23]. The most obvious difference between our results and this prior study was that the previous study included both urban and rural subjects, whereas we collected data only in an urban setting. It is reasonable to assume that information bias could affect the result. HCV is transmitted mainly through contact with blood and blood products, and this includes blood transfusions. However, with the advent of routine blood screening for HCV antibodies (in 1991 in most countries), transfusion-related hepatitis C has almost disappeared. In developing countries, routine blood screening is better available in urban than rural settings.

As in other studies [28-30], we identified illegal intravenous drug use as a mode of HCV transmission. Nevertheless, the small number of subjects who admitted intravenous drug use makes definitive conclusions difficult. Intravenous drug use could not explain the prevalence of HCV antibodies in the study population.

The absence of positive young children is consistent with mother-to-child transmission being rare in Madagascar as described previously [23] and more generally in the epidemiology of HCV [14].

Detection of serum anti-HCV antibodies is indicative of past or active infection. HCV viraemia, as assessed by RT-PCR, is a much more accurate indicator of chronic hepatitis due to virus [31]. We found a prevalence of active HCV infection of 0.8% in the study population, lower than the 5.7% reported for Morocco population [32] and the 2.3% reported in Sudan [33]. More than half the isolates (52.9%) were subtype 1b. Genotype 1b is also the most prevalent in many other countries [34]. Note that genotype 1b is less responsive than genotypes 2 and 3 to alpha-interferon therapy [34]. However, incomes in Madagascar are so low that few patients can afford treatment.

## Conclusion

In conclusion, these results for an urban population in Antananarivo indicate that the prevalence of HCV has not increased since a previous study in Madagascar [23]. Our findings suggest that the rate of HCV infection in Madagascar has been low and raise questions about the reservoir levels and iatrogenic transmission. This study contributes to our understanding of the worldwide prevalence of hepatitis C, which in turn facilitates informed decisions regarding the priorities of funding for the treatment, prevention and surveillance of this infection. Research in this field will need to be continued and in particular targeting rural populations. The incidence of the virus elsewhere in Madagascar should be evaluated, and the transmissibility of the various genotypes should be assessed. Better prevention, screening and treatment methods for Hepatitis C virus all need to be identified and described. However, in view of our findings, there is a need to educate general population regarding HCV infection and the risks associated with inappropriate therapeutic injections.

## Competing interests

The author(s) declare that they have no competing interests.

## Authors' contributions

RCE, RF, RM, AV, SJL conceived the study and participated in its design and coordination; RV, RR^2^, RR^1 ^were investigators for the study, participated in the design of the study, supervised the acquisition of the data, and helped analyze data; RAM, RD carried out laboratory analysis and helped analyze data; VR revised the manuscript and is the corresponding author; RLP is the principal investigator of the study and led the drafting of this manuscript.

All authors read and approved the final manuscript.

## Pre-publication history

The pre-publication history for this paper can be accessed here:


